# Cytotoxicity of Selected Novel Chalcone Derivatives on Human Breast, Lung and Hepatic Carcinoma Cell Lines 

**Published:** 2014

**Authors:** Maryam Nakhjavani, Afshin Zarghi, Farshad H. Shirazi

**Affiliations:** a *Department of Pharmaco-Toxicology, Shahid Beheshti University of Medical Sciences, Tehran, Iran. P.O.Box 14155-6153. *; b *Department of Medicinal Chemistry, Shahid Beheshti University of Medical Sciences, Tehran, Iran. P.O.Box 14155-6153. *; c *Pharmaceutical Sciences Research Center, Shahid Beheshti University of Medical Sciences, Tehran, Iran. P.O.Box 14155-3817. *

## Abstract

Cancer is considered as a challenging deathly disease and discovering or synthesis of new cytotoxic agents is a worldwide attempt. In this study, a group of recently synthesized chalcones, with the structure of 1,3-diarylprop-2-en-1-one having different COX-1 and/or COX-2 selectivities have been examined on human hepatocarcinoma (HepG2), lung carcinoma (A549), and breast adenocarcinoma (MCF-7) cell line, using Sulforhodamine B (SRB) assay. Briefly, cells were treated with 1-100 μM of each compound for 72, 96 and 168 hours. In each case, a control row was set with the exposure of cells to compounds-free solvents. Median lethal concentration (LC_50_) values (compared to controls) were calculated using regression fitness analysis on GraphPad Prism^®^ software. Our results show that the subgroup possessing *p*-azido COX-2 pharmacophore seems to be more cytotoxic, while the cells seem to show more acquired resistance to them and the subgroup possessing a *p*-MeSO2NH COX-2 pharmacophore is less cytotoxic, while the cells also acquire less resistance to them. In conclusion, considering the diversity in COX-1 or COX-2 inhibition among these compounds in each group, and also revealing no correlation between COX inhibition selectivity and cell death, it seems that selective inhibition of each isoenzyme doesn’t cause substantial effect on toxicity potency. Further studies to determine the main mechanism(s) for these compounds induced cell death are encouraged.

## Introduction

Novel compounds are the hope in cancer chemotherapy. Chalcone compounds are among the leading compounds for cancer chemotherapy with proposed anti-COX2 activities (1). Cytotoxicity assay of newly prepared agents is a “must” which is the focus of this study. Chalcones, with chemical structures of an aromatic ketone and an enone having a central core, have become a favorable candidate for cancer therapy of different origins. They have been shown to have anti-tumorigenic activities (2, 3). Different cellular and molecular mechanisms have been proposed for various chalcones (4-6). Several chalcone structures have been synthesized and tested on human carcinoma cell lines with promising anti-proliferative results at micromolar to nanomolar concentrations (7, 8). However, less is known about the effects of these compounds on carcinoma cell lines and cells reactions to these compounds by the time of exposure.

Besides the role of cyclooxygenase-2 (COX-2) in inflammation, it has been shown that this isoenzyme plays roles in several cancers (9). Zarqhi *et al*., in a study showed that chalcones with the chemical structure of 1,3-diphenylprop-2-en-1-one constitute a suitable scaffold for selective COX-2 inhibitory activity (10), which seems to be a promising target enzyme for cancer treatment (11, 12). In fact, a large amount of epidemiological and experimental evidences support a role for COX-2 in oncogenesis (13, 14). It is now recognized that COX-2 over expression promotes tumorigenesity, which can be suppressed by NSAIDs and COX-2 inhibitors, a phenomenon useful for the suppression of tumor progression (15).

In the present study, the cytotoxicity of a group of novel 1,3-diphenylprop-2-en-1-one chalcones are determined on three COX-2 expressing cell lines; human breast MCF-7 adenocarcinoma (16), human lung A549 adenocarcinoma (17), and human HepG2 hepatocarcinoma (18). Generally, COX-2 is highly expressed in aggressive metastatic breast cancers (*i.e. *growth and metastasis) (19) and in lung carcinoma (20). Some reports have also shown that NSAIDs inhibit the growth of hepatocellular carcinoma cell lines, a rational to suggest their benefit in liver cancer therapy to be associated with liver carcinogenesis (21-23). To define the characteristics of the compounds-induced cytotoxicity and time based cellular response to these effects, we conducted the present study testing the cytotoxicity of each compound at 72, 96 and 168 hours exposure time, using Sulforhodamine B (SRB) assay, which is an anionic dye that binds to intracellular proteins fixed in the plate and thus provides a sensitive index of cell viability (24).

## Experimental

*Materials*

Compounds, otherwise specified, were purchased from Sigma^®^.

*Chemicals*

Different 1,3-diarylprop-2-en-1-one derivatives (Table 1) were synthesized in the department of medicinal chemistry of the school of pharmacy, Shaheed Beheshti university of medical sciences, as previously explained (25). Briefly, 1,3-diphenylprop-2-en-1-ones with an azido, or methansulfonamido substituent attached to the C-1 phenyl ring were prepared by one-step Claisen-Schmidt condensation.

**Figure:**
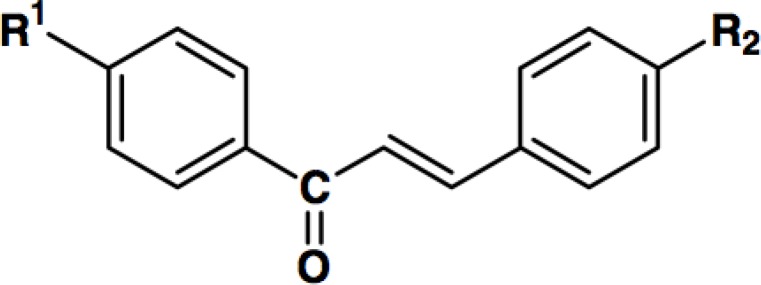


**Table 1:** Chemical structure of different 1,3-diphenylprop-2-en-1-one derivatives with various substitutions.

Compound	R1	R2
1	N_3_	H
2	N_3_	F
3	NHSO_2_Me	H
4	NHSO_2_Me	OMe
5	NHSO_2_Me	F

*Cell cultures*

Human HepG2 hepatocarcinoma cell line (ATCC No. HB8065), human lung carcinoma A549 cell line (ATCC No. CLL-185), and human breast adenocarcinoma MCF-7 cells (ATCC No. HTB-22), were purchased from the Iranian Cell Bank of Pasture Institute, thawed and propagated for three passages in DMEM/F12 (Dulbecco᾽s Modified Eagle Medium, Nutrient mixture F-12)(Gibco BRL^®^, USA) in flasks of 25 and 75 cm2 surfaces (Orange^®^, Canada). Cells were plated at 6 × 10^3^ cells per well for HepG2 and A549 and 3 × 10^3^cells per well for MCF-7 in 96-well flat-bottomed plates (Orange^®^, Canada) containing 180 uL of medium.

*Cytotoxicity tests*

The synthesized compounds were evaluated using the following protocol on above mentioned cell lines. The compounds were dissolved in dimethylsulfoxide (DMSO) as stoke to make the final concentrations of 0-100 μg/mL by serial dilutions. Cisplatin as the positive control was dissolved in normal saline and further concentrations of 0-20 μg/mL were prepared by serial dilutions. Following overnight incubation of cells, 20 uL of each concentration was added to each well (final concentration of DMSO < 1%), control cultures received equivalent volumes of DMSO in the medium. All cultures were incubated at 37 ˚C in a 95% air/5%CO_2_ humidified incubator. After 72, 96, or 168 hours of exposure, cell viability was determined by the protein binding SRB assay, according to the below protocol, for each well. The supernatant of each well was removed and adherent cells were fixed with 20% (w/v) cold trichloroacetic acid at 4 °C. After 30 minutes, the cells were washed with deionized distilled water and were air-dried. 50 μL of Sulforhodamine B (0.4% (w/v) in 1% acetic acid) was exposed to cells for 30 minutes, and cells were washed with 1% acetic acid and air dried. Finally, 200 μL of 10 mM unbuffered Tris (pH=10) was added to each well and the plates were shaken for 30 minutes. The optical density was measured at 540 nm.

*Statistics*

The median lethal concentration (LC_50_) of each compound was calculated by plotting the percent of cell survivals vs concentrations of the test compounds using nonlinear regression analysis in GraphPad PRISM^®^ Software. All comparisons were performed in SPSS^®^ software using General Linear Model, repeated measurement test. Comparison p values are less than 0.05 for all comparisons mentioned in the result sections and thus are not mentioned within the texts for the sake of simplicity.

## Results and Discussion

Two different groups of compounds have been evaluated for cytotoxicity on cancer cells in this study which physic-chemical characteristics are presented in Table 2. The COX inhibition properties and cytotoxicities are presented below.

**Table 2:** Physico-chemical properties of compounds 1-5

**Compound**	**Color and Crystal Form**	**Melting Point (°C)**	**Yield (%)**	**Formula**
1	Cream crystalline powder	115-116	72	C_15_H_11_NO_3_
2	Cream crystalline powder	138-139	70	C_15_H_10_FNO_3_
3	Yellow crystalline powder	169-170	40	C_16_H_15_NO_3_S
4	Yellow crystalline powder	132-134	44	C_17_H_17_NO_4_S
5	Yellow crystalline powder	192-194	50	C_16_H_14_FNO_3_S

*The subgroup of 1,3-diphenylprop-2-en-1-ones possessing a C-1 p-azido COX-2 pharmacophore*

As previously described, these compounds exhibited good COX-2 inhibitory potency and selectivity, as shown in Table 3. Compound 1 shows moderate COX-2 inhibition with no inhibition of COX-1 at 100 μM. On the other hand compound 2, which could inhibit both COX-1 and COX-2, has been shown to be 2.5 fold more potent inhibitor of COX-1 (25). 

**Table 3:** In-vitro COX-1 and COX-2 enzyme inhibition assay data for 1,3-diphenylprop-2-en-1-one derivatives 1-5.

**Compound**	**IC**_50_ a		**Selectivity Index (SI)** ^b^	
	**COX-1**	**COX-2**		
1	>100	3.4	>29
2	4.2	10.0	0.4
3	3.0	3.2	0.9
4	1.0	10.0	0.1
5	3.3	>100	-

a Values are means of two determinations acquired using an ovine COX-1/COX-2 assay kit (catalog no. 560101, Cayman Chemicals Inc., Ann Arbor, MI, USA) and the deviation from the mean is <10% of the mean value.

b *In-vitro *COX-2 selectivity index (COX-1 IC50/COX-2 IC50).

**Figure 1:**
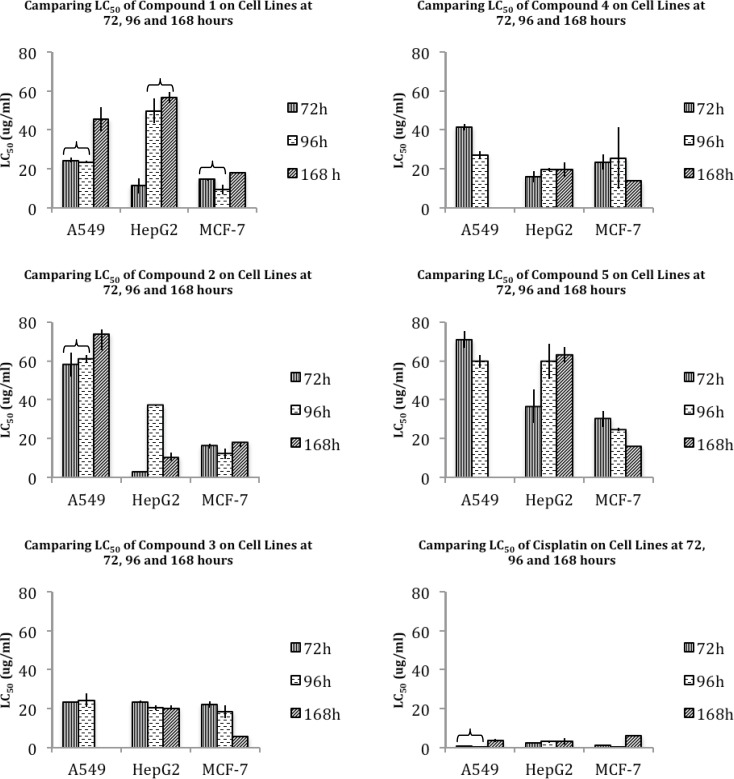
Comparing median lethal concentration (LC_50_) of compounds 1-5 and cisplatin on human lung A549, hepatic HepG2 and breast MCF-7 carcinoma cell lines, at 72, 96 and 168 hours. p-value < 0.05.

Various studied cell lines seem to reveal similar patterns of cytotoxicity for these two compounds. An exposure time-dependent decrease in cytotoxicity is obvious for lung A549, hepatic HepG2, and breast MCF-7 cells with the exception of unusual cytotoxic pattern of HepG2 exposed to compound 2. Generally, from the cytotoxicological point of view, lung A549 cells seem to be less sensitive to these compounds, while breast MCF-7 cells seem to be the most sensitive cell line (Figure 1). This sensitivity is well noticeable especially at 96 hours of exposure to compounds 1 and 2 (Table 4), which are comparable to the well-known chemotherapeutic agent cisplatin that is widely applicable in many cancers (26). However, it is important to notice that no statistically significant difference is obvious between the cytotoxicity induced by compound 1 or 2, suggesting that COX selectivity might not be the dominant mechanism of cell death induced by these compounds.

**Table 4:** Median lethal concentration (LC_50_) amounts ± standard deviation (SD) of compounds 1-5 and cisplatin on A549, HepG2 and MCF-7 cell lines, at 72, 96 and 168 hours

**Cell line**	**A549**			**HepG2**			**MCF-7**		
	**72 h**	**96 h**	**168 h**	**72 h**	**96 h**	**168 h**	**72 h**	**96 h**	**168 h**
1	24.31 ± 1.30	23.42 ± 0.64	45.5 ± 6.17	11.19 ± 4.07	49.7 ± 6.42	56.52 ± 2.86	14.56 ± 0.69	9.482 ± 2.48	17.92 ± 0.19
2	58.09 ± 6.21	61.07 ± 1.85	73.95 ± 8.34	2.95 ± 0.19	37.09 ± 0.38	10.34 ± 2.03	16.12 ± 1.39	12.31 ± 2.65	18.14 ± 2.55
3	23.47 ± 0.31	24.06 ± 3.57	ND^a^	23.43 ± 0.61	20.55 ± 1.26	20.21 ± 1.42	22.12 ± 1.59	18.4 ± 3.30	5.60 ± 1.29
4	41.42 ± 1.80	27.09 ± 1.95	ND	15.85 ± 2.84	19.63 ± 0.80	19.73 ± 0.81	23.30 ± 3.97	25.52 ±15.9	13.73 ± 3.73
5	70.96 ± 4.16	59.74 ± 3.22	ND	36.61 ± 8.53	59.75 ± 8.96	62.96 ± 6.85	30.19 ± 4.14	21.14 ± 3.07	15.98 ± 4.03
Cisplatin	0.81 ± 0.31	0.008 ± 0.005	3.38 ± 0.86	2.20 ± 0.20	3.03 ± 0.57	3.21 ± 1.56	0.96 ± 0.27	0.04 ± 0.12	6.14 ± 0.04

a : Not Determined.

*3-2-The subgroup of 1,3-diphenylprop-2-en-1-enes possessing a C-1 p-MeSO2NH COX-2 pharmacophore*

In this group, compound 3 with an unsubstituted C-3 phenyl ring showed to have equipotent nonselective inhibition of COX-1 and COX-2, while substituting a *p*-OMe-phenyl resulted in a higher COX-2 inhibition (compound 4). On the other hand, C-3 *p*-fluoro substitution decreased the COX-2 inhibition and increased COX-1 inhibition as well as the total potency of compounds (especially in compound 5) (Table 3) (25).The general pattern of cytotoxicity of these compounds seems to be similar in most studied cases. Time does not seem to influence the cytotoxicity pattern of HepG2 in any cases, while an increase in cytotoxicity of long-term exposure is observable for A549 and MCF-7 (compounds 4 and 5) (Figure 1). In this group of compounds also, MCF-7 seems to be the most sensitive cell line. However, presenting compound 3 as the most cytotoxic agent claims again that selective COX-1 or COX-2 inhibition may not be considered as the main mechanism of cell death induced by these compounds.

## Conclusion

In conclusion, the subgroup of 1,3-diphenylprop-2-en-1-ones possessing a C-1 *p*-azido COX-2 pharmacophore showed higher cytotoxicity, although the understudied cells have later shown more acquired resistance to these compounds. On the other hand, although the subgroup with a C-1 *p*-MeSO_2_NH COX-2 pharmacophore is less cytotoxic, however, cells show less resistance to these compounds It is valuable to notice that all of these compounds share different amounts of COX-1 and COX-2 selectivity. Considering the diversity in COX-1 or COX-2 inhibition among these compounds in each group, and also revealing no correlation between COX inhibition selectivity and cell death, it seems that selective inhibition of each isoenzyme does not cause substantial effect on their toxicity potency. Despite COX-1 and/or COX-2 inhibition induced by these compounds, structure dependent cytotoxicity changes of these compounds, is independent to COX-1 and/or COX-2 inhibition. Therefore, further studies to investigate and determine the main mechanism(s) of cell death induced by these compounds and their related mechanims for each compound is valuable and encouraged. 
